# Prostate cancer risk prediction using a polygenic risk score

**DOI:** 10.1038/s41598-020-74172-z

**Published:** 2020-10-13

**Authors:** Csilla Sipeky, Kirsi M. Talala, Teuvo L. J. Tammela, Kimmo Taari, Anssi Auvinen, Johanna Schleutker

**Affiliations:** 1 Institute of Biomedicine, University of Turku, Kiinamyllynkatu 10, 20520 Turku, Finland; 2 Finnish Cancer Registry, Mass Screening Registry, Helsinki, Finland; 3 Department of Urology, Tampere University Hospital and Faculty of Medicine and Health Technology, Tampere University, Tampere, Finland; 4 Department of Urology, University of Helsinki and Helsinki University Hospital, Helsinki, Finland; 5 Unit of Health Sciences, Faculty of Social Sciences, Tampere University, Tampere, Finland; 6 Department of Medical Genetics, Genomics, Laboratory Division, Turku University Hospital, Turku, Finland

**Keywords:** Biomarkers, Oncology, Risk factors, Urology

## Abstract

Hereditary factors have a strong influence on prostate cancer (PC) risk and poorer outcomes, thus stratification by genetic factors addresses a critical need for targeted PC screening and risk-adapted follow-up. In this Finnish population-based retrospective study 2283 clinically diagnosed and 455 screen-detected patients from the Finnish Randomised Study of Screening for Prostate Cancer (FinRSPC), 2400 healthy individuals have been involved. Individual genetic risk through establishment of a polygenic risk score based on 55 PC risk SNPs identified through the Finnish subset of the Collaborative Oncological Gene-Environment Study was assessed. Men with PC had significantly higher median polygenic risk score compared to the controls (6.59 vs. 3.83, *P* < 0.0001). The polygenic risk score above the control median was a significant predictor of PC (OR 2.13, 95% CI 1.90–2.39). The polygenic risk score predicted the risk of PC with an AUC of 0.618 (95% CI 0.60–0.63). Men in the highest polygenic risk score quartile were 2.8—fold (95% CI 2.4–3.30) more likely to develop PC compared with men in the lowest quartile. In the FinRSPC cohort, a significantly higher percentage of men had a PSA level of ≥ 4 ng/mL in polygenic risk score quartile four compared to quartile one (18.7% vs 8.3%, *P* < 0.00001). Adding the PRS to a PSA-only model contributed additional information in predicting PC in the FinRSPC model. Results strongly suggest that use of the polygenic risk score would facilitate the identification of men at increased risk for PC.

## Introduction

Although prostate cancer (PC) remains the second most common cancer in men, biomarkers for accurate early diagnosis are missing^1,2^. Owing to the well-known and inherent limitations of using prostate-specific antigen (PSA), novel biomarkers to complement PSA for the prediction of PC and particularly PC with unfavourable outcomes are needed^3^.

Prostate cancer has a very high heritability, with an estimate of 57% (95% CI 0.51–0.63) based on the Nordic Twin Study of Cancer^4^, with only a few identified high-risk genes. High penetrance genes, like *BRCA1/2*, *HOXB13*, *CHEK2* and *MMR* are important, but explain only a fraction of inherited PC risk^5^. In addition, the nearly 170 single nucleotide polymorphisms (SNPs) associated with PC risk in large genome-wide studies explain a quarter of the familial risk, and offer an opportunity to create a polygenic risk score (PRS) to identify the subgroups of the population at highest risk of the disease^6,7^. Combining a broad array of genetic data into a PRS offers an opportunity for accurate prediction of PC risk^8^ and allows to highlight the sharp contrast in probability of developing the disease between the highest and lowest risk groups^9^.

There is evidence of population stratification, i.e. differences between populations in the genetic factors contributing to familial PC risk. Previous studies have estimated PRSs mainly based on pooled data from multinational cohorts. Instead of using results from mixed European populations, we utilized population-specific PC risk loci identified from the Finnish subset from Collaborative Oncological Gene-Environment Study (iCOGS)^8^.

The aim of our study was to evaluate the potential of PRS to predict PC risk in advance and its application in personalized PC diagnosis*.* Specifically, we evaluated the accuracy of genetic risk stratification to predict overall PC, and separately for clinically diagnosed patients and screening trial cases. Moreover, the aim was to assess the ability of PRS to predict PC subgroups defined by clinical parameters such as high PSA at diagnosis, aggressive disease and advanced stage. In the Finnish Randomised Study of Screening for Prostate Cancer (FinRSPC) cohort, we evaluated the additional contribution of PRS to PSA and age.

## Results

### Prostate cancer risk

The median PRS in men with PC was 6.59 (interquartile range (IQR) 8.29) versus 3.83 (IQR 8.02) among cancer-free controls (*p* < 0.0001) (Table 1). There was no statistically significant difference in the median PRS of men with clinically detected (PRS 6.74, IQR 8.41) vs screen-detected (PRS 6.31, IQR 7.14) cancer.

**Table 1:** Polygenic risk score of men predicting prostate cancer in Finland*.

SAMPLE GROUPS	N	Mean (95% CI)	St error of mean	Median	Variance	Standard deviation
Study population	5138	5.99 (5.83–6.16)	0.085	5.01	37.05	6.09
Men without prostate cancer (controls) *(FinRSPC)*	2400	4.71 (4.47–4.94)	0.119	3.83	33.68	5.80
Men with prostate cancer (cases)	2738	7.12 (6.89–7.35)	0.117	6.59	37.28	6.11
Screening trial cases *(FinRSPC)*	455	6.65 (6.09–7.20)	0.282	6.31	36.22	6.02
Clinical cases *(Pirkanmaa Hospital)*	2283	7.22 (6.97–7.47)	0.128	6.74	37.45	6.12

*Polygenic risk score is based on 55 prostate cancer susceptibility loci.

Of all PC cases, 68.2% had a PRS above the population control’s median (3.83) (Table 2), corresponding to a sensitivity of 0.68 (95% CI 0.66–0.70) and specificity of 0.50 (0.48–0.52). The positive predictive value was 0.61 (95% CI 0.59–0.63) and positive likelihood ratio 1.36 (95% CI 1.30–1.43). Out of men with a clinically diagnosed PC, the proportion was 68.7%, while for the screening trial cases 65.9%. The odds ratio for overall PC with a PRS above the control median was 2.13 (95% CI 1.90–2.39), the corresponding OR for clinical PC was 2.18 (95% CI 1.93–2.45) and for screening trial cases was 1.92 (95% CI 1.56–2.37).

**Table 2:** Evaluation of polygenic risk score to predict prostate cancer risk and associated clinical measures.

	Logistic regression			Receiver Operator Characteristics	
	High PRS N (%)	OR (95% CI)	P value	AUC (95% CI)	P value
***Risk of prostate cancer:***					
All prostate cancers (n = 2738)	1868 (68.2)	**2.13 (1.90–2.39)**	**5.92E-39**	**0.618 (0.60.0.63)**	**4.79E-48**
Clinical prostate cancer cases (n = 2283)	1568 (68.7)	**2.18 (1.93–2.45)**	**1.49E-37**	**0.622 (0.61–0.64)**	**4.57E-47**
Screening trial prostate cancer cases (n = 455)	300 (65.9)	**1.92 (1.56–2.37)**	**1.11E-9**	**0.597 (0.57–0.63)**	**4.42E-11**
^***1***^***Risk of prostate cancer with:***					
High PSA at diagnosis (> 20 ng/mL) (n = 484)	339 (70.0)	1.12 (0.90–0.39)	0.300	0.517 (0.49–0.54)	0.232
^2^Aggressive (Gleason score ≥ 8) (n = 368)	235 (63.9)	0.83 (0.65–1.06)	0.140	0.478 (0.44–0.51)	0.206
^3^Advanced stage (n = 602)	418 (69.4)	1.08 (0.89–1.32)	0.425	0.517 (0.48–0.55)	0.346
Death by prostate cancer (n = 304)	204 (67.1)	0.94 (0.73–1.22)	0.657	0.497 (0.46–0.53)	0.865
Tumour stage (T3-T4) (n = 540)	369 (68.3)	1.01 (0.81–1.25)	0.936	0.512 (0.48–0.54)	0.435
Cancer in nodus (N1 stage) (n = 14)	9 (64.3)	0.84 (0.28–2.51)	0.752	0.443 (0.30–0.58)	0.462
Metastasis (M1 stage) (n = 191)	144 (75.4)	**1.47 (1.04–2.06)**	**0.028**	**0.549 (0.51–0.59)**	**0.023**

^1^case only investigation; ^2^versus Gleason score ≤ 6; ^3^advanced disease stage is defined as T3-4, or N1 or M1; OR, odds ratio (polygenic risk score above median was applied as binary variable); AUC, area under the curve (polygenic risk score was used as continuous variable); statistically significant results are indicated with bold.

When divided into PRS quartiles, PC cases were distributed 18%, 25%, 27% and 30% from the lowest to the highest quartile. For the controls, the proportions showed an opposite pattern (33%, 26%, 22%, 19%, respectively). Showing that nearly a third of the PC cases belong to the highest PRS quartile, while one-third of the controls belong to the lowest PRS quartile. Men in the highest PRS quartile were of 2.8—fold (95% CI 2.40–3.30) higher risk of PC compared with men in the lowest quartile.

The overall receiver operator curve AUC of the PRS to predict PC was 0.618 (95% CI 0.60–0.63, p 4.79E-48), for clinically diagnosed PC was 0.622 (95% CI 0.61–0.64, p 4.57E-47), and for screening trial PC cases was 0.597 (95% CI 0.57–0.63, p 4.42E-11) (Table 2).

### Prostate cancer clinical parameters

Of the patients with metastatic PC, 75.4% had a PRS above the control median, corresponding to an OR of 1.47 (95% CI 1.04–2.06, p 0.028, Table 2) with an AUC of 0.549 (95% CI 0.51–0.59). Although, 70.0% of the men with high PSA at diagnosis (PSA > 20 ng/mL) had a PRS above the median, no association between the PRS and high PSA at diagnosis could be identified. Similarly, there was no significant association between PRS and high Gleason score, advanced stage, tumour and nodal stage or lethal PC, possibly due to the nature of the SNPs included and low amount of cases (Table 2).

Further, there was no statistically significant association between the quartiles of the PRS and age at onset of PC (χ^2^ = 3.15; *p* = 0.369), PSA at diagnosis (χ^2^ = 3.58; *p* = 0.311), Gleason score (χ^2^ = 5.37; *p* = 0.147) or disease stage (χ^2^ = 1.41; *P* = 0.703; Supplementary Table 1).

### Association with PSA in the subset of FinRSPC cohort: the FinRSPC model

When the FinRSPC cohort was divided into negative and positive PSA (PSA < 4 ng/mL vs PSA ≥ 4 ng/mL), the number of men with elevated PSA increased in each PRS quartile (Table 3, A). The association between PSA and PRS is illustrated by the fact that 8.3% of men in the lowest PRS quartile had PSA ≥ 4 ng/mL compared to 18.7% in the highest quartile (χ^2^ = 32.95; *P* < 0.00001).

**Table 3:** Evaluation of polygenic risk score in prostate cancer screening in the subset of FinRSPC cohort.

PRS	PSA < 4 ng/mL		PSA ≥ 4 ng/mL		% of men with PSA ≥ 4 ng/mL in different quartiles (*p* < 0.00001)
	N	%	N	%	
*A) Association between quartile of polygenic risk score and PSA*					
Q1 (n = 713)	654	27	59	15	8.3%
Q2 (n = 711)	623	25	88	22	12.4%
Q3 (n = 717)	592	24	125	31	17.4%
Q4 (n = 707)	575	24	132	33	18.7%

Risk factor	SIMPLE MODEL		MULTIPLE MODEL		
	OR (95% CI, p)	AUC (95% CI, p)	OR (95% CI)	AUC cumulative (95% CI, p)	
*B) Simple and multiple logistic regression models including PSA, age, polygenic risk score and their predictive performance to predict prostate cancer*					
PSA	6.50 (5.43–7.80, 2.61E-90)	0.981 (0.97–0.99, 3.17E-232)	6.60 (5.48–7.95, 8.29E-28)	0.982 (0.97–0.99, 2.70E-232)	
Age	1.04 (1.01–1.07, 0.016)	0.557 (0.53–0.59, 0.000116)	1.02 (0.94–1.11, 0.683)	0.557 (0.53–0.59,0.000116)	
PRS	1.06 (1.04–1.07, 1.59E-10)	0.597 (0.57–0.63, 4.42E-11)	1.05 (1.00–1.11, 0.038)	0.600 (0.57–0.63, 1.80E-11)	

P value based on χ^2^ test; PRS, polygenic risk score; PSA, prostate-specific antigen; Q, quartile.

OR = odds ratio; CI = confidence interval; AUC = area under the receiver operating characteristics curve; PRS, polygenic risk score; Predictive performance was assessed using the AUC for individual risk factors and for the genetic model including all risk factors. AUC cumulative denotes AUC values obtained when one risk factor at the time was added to the model from PSA only to a model including all risk factors. PSA, Age, PRS were used as continuous variables. Men aged 55–67 years at the enrollment to the FinRSPC trial.

In unadjusted logistic regression within the FinRSPC cohort, both PSA (OR 6.50, 95% CI 5.43–7.80), PRS (OR 1.06, 95% CI 1.04–1.07) and age (OR 1.04, 95% CI 1.01–1.07) predicted the risk of PC (Table 3, B). After mutual adjustment, PRS (OR 1.05, 95% CI 1.00–1.11) was still associated with PC risk, indicating that it contributed additional information beyond that provided by PSA.

## Discussion

We constructed a population-specific PRS for PC and evaluated its application in genetic risk stratification. The Prs was higher among men with PC than the controls, as indicated by the median and proportion above the control median, with an odds ratio of 2.13. The AUC was 0.62, with sensitivity of 0.68. The PC risk also increased with PRS when it was divided into quartiles. The PRS was associated with metastatic disease, however, it was not associated with other indicators of poor prognosis such as high Gleason score or advanced disease. Furthermore, within the screening trial, PRS was associated with the proportion of men with positive PSA^10^ and contributed to detect PC.

Our finding (ROC 0.62) was comparable with previous studies, despite our use of a relatively small number of SNPs (n = 55). Previous studies using risk allele based polygenic scores have shown ROC values of 0.54–0.68 for PC^11–13^. However, they have provided only limited evidence that the PRS using common variants improves risk prediction^8,12,13^.

Genome-wide association studies (GWASs) for metastatic PC are lacking^14^ and few studies have investigated the association between known germline PC risk variants and metastatic disease diagnosis or development of metastasis after initial treatment^14,15^. All of the metastatic patients in this cohort had already been found at diagnosis due to the retrospective nature of this study. The identified association of PRS with metastatic disease at diagnosis is likely due to the inclusion of PC risk SNPs, which have earlier been found to be associated with metastatic PC risk^14–16^. The lack of association with other clinical variables is in line with earlier findings. Since the performance of our PRS was poorer for metastatic disease than PC overall, it offers only limited use for individual prediction of the risk of metastasis.

PSA has long been used as the primary biomarker for PC diagnosis, however, PSA screening results are frequent in false-positive results and overdiagnosis^17,18^. Therefore, population-based screening is not recommended^19,20^. In this study, we show that in the FinRSPC screening cohort the PRS quartiles are associated with elevated PSA of ≥ 4 ng/mL at diagnosis and that the PRS contributed additional information beyond PSA and age in predicting PC in the screening trial men. Performance of the PRS in screening needs an additional prospective cohort in order to test its applicability for population-based screening to supplement PSA-based stewardship in screening for PC.

The main strength of the study is that it is population-based, therefore the selection bias is minimized and the generalizability is increased. Previous studies have included mainly risk variants (OR > 1) for the construction of the PRS^13^. We used both risk (per allele OR > 1) and protective (per allele OR < 1) SNPs to capture genetic variation in risk more widely. Furthermore, we designed a population-specific risk score, as PC risk variants and their frequencies differ between populations^21^.

Naturally, the study has some limitations. In the study population there are only few PC deaths and aggressive cases. Since it is based on retrospective data, validation in a prospective setting will elevate the power and would potentially improve the study. In particular, application of the PRS in population screening needs to be conclusively evaluated in a prospective trial in order to test the PRS for clinical implications and potential benefit. Since this a Finnish population based PRS study, application in other, less homogeneous population is needed.

Our findings show that a subgroup of men at an increased risk of PC (OR > 2) can be identified based on a PRS. However, the accuracy in predicting was limited (AUC 0.62). The fact that PRS contributed additional information above PSA and age suggests that its usefulness in screening is worthwhile.

## Materials and methods

All methods were carried out in accordance with relevant guidelines and regulations.

**Figure 1:**
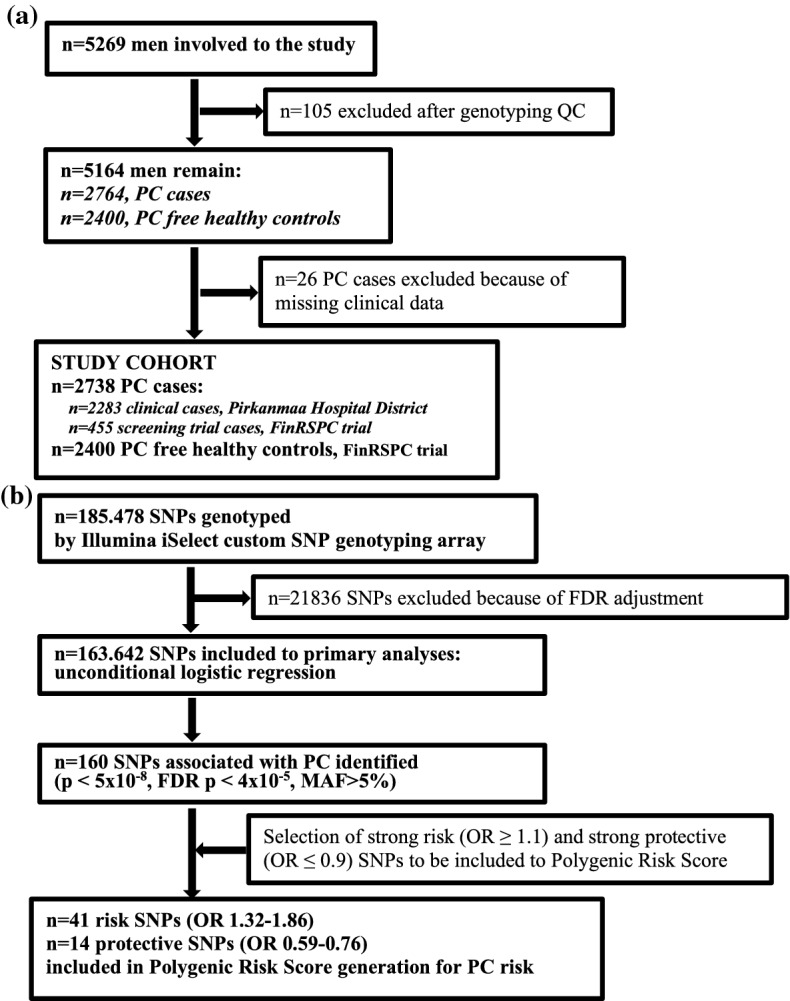
Flow diagram presenting the steps of participant's enrollment to the study (**a**) and selection of SNPs for polygenic risk score (**b**) QC, quality control; FDR, false discovery rate, corrected by Benjamini–Hochberg method.

The flow diagram shown in Fig. 1 presents the steps of participant enrolment to the study (A) and selection of SNPs for PRS calculation (B).

### Study participants

All genotyped PC patients and controls without PC were of Finnish origin. The study protocol was reviewed and approved by the research Ethics committee at Pirkanmaa Hospital District (tracking numbers R10167, 90,577, R03203). Permission for the use of samples was given by the National Supervisory Authority for Welfare and Health (VALVIRA). Informed consent was obtained from the participants involved in the study. Altogether, 2738 non-familial PC cases were included in the study. Of them, 2283 were clinical cases from the Pirkanmaa Hospital District, and 455 were from the Finnish Randomized Study of Screening for Prostate Cancer (FinRSPC)^22^, which is the largest component of the European Randomized Study of Screening for Prostate Cancer (ERSPC)^23^. Cancer free control subjects (n = 2400) were identified through the FinRSPC trial^22^. The FinRSPC trial population and the protocol population have been described in detail elsewhere^24^. Briefly, 80,458 men aged 55–67 years were enrolled during 1996–1999, with 32,000 randomised to the screening arm and invited to PSA-based screenings at four-year intervals.

Clinical characteristics of the genotyped PC patients, separately for clinically detected and for screening trial cases, are summarized in Table **4**. PSA at diagnosis was classified as ≤ 20 versus > 20 ng/mL. Gleason score was divided into ≤ 6, 7 and ≥ 8. Stage was divided into organ-confined (T1-2, N0/x, M0/x) versus advanced disease (T3-4, or N1 or M1). PC death was defined based on the underlying cause recorded as the official cause of death by Statistics Finland.

**Table 4:** Baseline clinical characteristics of non-familial prostate cancer patients.

Clinical variables	Clinical cases (n = 2283)	Screening trial cases (n = 455)
**Age at diagnosis**		
Early onset (≤ 55 y)	103 (4.50)	3 (0.70)
Late onset (> 55 y)	2180 (95.5)	452 (99.3)
**Diagnostic PSA level, ng/mL**		
Low, ≤ 20	1684 (73.8)	415 (91.2)
High, > 20	455 (19.9)	29 (6.40)
Missing data	144 (6.30)	11 (2.40)
**Gleason score**		
Low, ≤ 6	1033 (45.3)	287 (63.1)
High, ≥ 8	329 (14.4)	39 (8.60)
Gleason 7	564 (24.7)	121 (26.6)
Missing data	357 (15.6)	8 (1.80)
**T stage**		
T0/Tx	0 (0.00)	13 (2.90)
T1	746 (32.8)	359 (78.9)
T2	922 (40.3)	50 (11.0)
T3	418 (18.3)	25 (5.50)
T4	96 (4.20)	1 (0.20)
Missing data	101 (4.40)	7 (1.50)
**N stage**		
N0/Nx	2169 (95.0)	447 (98.2)
N1	13 (0.60)	1 (0.20)
Missing data	101 (4.40)	7 (1.50)
**M stage**		
M0/Mx	1999 (87.6)	440 (96.7)
M1	183 (8.00)	8 (1.80)
Missing data	101 (4.40)	7 (1.50)
**PSA Progression**		
Progressed	804 (35.4)	156 (34.3)
Missing data	1479 (64.8)	299 (65.7)
**Death**		
Deceased of PC	296 (13.0)	8 (1.76)
Deceased of else	864 (37.9)	63 (13.9)
Alive	1123 (49.2)	384 (84.4)

### Genotyping and quality control

The original genotyping was carried out by the PRACTICAL (Prostate Cancer Association group to Investigate Cancer Associated Alterations in the Genome) consortium. The genotyping outcome was obtained from the use of a custom Illumina Infinium array (iCOGS), as described previously^8^.

### Single nucleotide polymorphism selection and statistical analyses

The Hardy–Weinberg equilibrium was ensured by checking that the proportion of each genotype obtained was in agreement with the expectation calculated from the allele frequencies. Statistical analyses were performed with IBM SPSS version 25 (SPSS Inc., Chicago, USA) unless otherwise specified. For each SNP, allelic ORs for PC with 95% confidence intervals were computed using logistic regression. A total of 55 variants shown to be associated with PC in the Finnish subset from iCOGS (Supplementary Table 2) were chosen for the calculation of the PRS based on the selection criteria described in Fig. 1. In short, selected SNPs were associated with PC at a genome-wide significance level (*p* < 5 × 10^–8^) and had the effect size of OR > 1.1 for risk SNPs and OR < 0.9 for protective SNPs.

We assessed the PRS of men with and without PC, and also separately for clinically diagnosed and screening trial cases. Sensitivity and specificity of the PRS were calculated. The use of the control median as the cut-off-point showed a near-optimal sensitivity and specificity. Therefore, the study participants were divided into those with a polygenic risk below and above the control median, which represents men free of PC. The odds ratio for PC risk prediction relating to the PRS above median was evaluated by logistic regression with PC as the outcome. We evaluated the predictive performance of PRS by calculating the area under the curve (AUC) of the receiver operator characteristic (ROC). Evaluation of the discriminative potential of the PRS for subsets of cases with high PSA at diagnosis, high Gleason score, advanced stage, local and distant progression, and PC death was performed with the same methodology.

In order to evaluate the possible implications of the PRS in the screening trial, we evaluated the additional contribution of PRS quartiles incremental to PSA and age in predicting PC in the FinRSPC cohort. Logistic regression models including PSA, age and the PRS were applied to assess PC prediction and the AUC calculated. All reported *p* values are two-sided.

### Polygenic risk score calculation

A PRS for each individual was calculated by summing the number of risk alleles^25^ at each of the 55 SNPs multiplied by the logarithm of the SNP’s OR as follows:

$$PRS_{j} = \mathop \sum \limits_{i = 1}^{n} \beta_{i} X_{ij}$$

where β*i* is the per-allele log-odds ratio for locus *i*, *x*_*ij*_ represents the number of risk alleles (i.e., 0, 1 or 2) carried by an individual *j* at locus *i*, and *n* is the number of loci. The risk conferred by each of the variants is assumed to be allele dose-dependent with a multiplicative (log-additive) effect on a relative risk scale^6^. Under the multiplicative model, the distribution of polygenic risk in the population follows the normal distribution, when relative risk is plotted on a logarithmic scale, with mean, μ, and variance σ^2^. We set the mean, μ = −σ^2^/2, so that the mean relative risk in the population is equal to unity. Log-transformation of non-normally distributed PRS data was applied.

## Supplementary information

Supplementary Information.
