# Supplementary material for: Prostate cancer risk prediction using a polygenic risk score
Source: Sci Rep. 2020 Oct 13;10:17075. doi: 10.1038/s41598-020-74172-z (PMC7553910; doi:10.1038/s41598-020-74172-z)
Supplement: Supplementary file 1 — Supplementary Information. [file 41598_2020_74172_MOESM1_ESM.pdf]

Article title: Prostate cancer risk prediction using a polygenic risk score

Authors: Csilla Sipeky, Kirsi M Talala, Teuvo L.J. Tammela, Kimmo Taari, Anssi Auvinen, Johanna Schleutker

Corresponding author contact information:

Johanna Schleutker, PhD
Professor of Medical Genetics
Institute of Biomedicine
University of Turku
Kiinamyllynkatu 10
20520 Turku, Finland
johanna.schleutker@utu.fi
Tel: +358 29 450 2726

SUPPLEMENTARY MATERIAL

The PRACTICAL Consortium:
Rosalind Eeles [1, 2], Doug Easton [3], Zsofia Kote-Jarai [1], Ali Amin Al Olama [3], Sara Benlloch [3], Kenneth Muir [4], Graham Giles [5, 6], Fredrik Wiklund [7], Henrik Gronberg [7], Christopher Haiman [8], Johanna Schleutker [9, 10], Maren Weischer [11], Ruth C. Travis [12], David Neal [13], Paul Pharoah [14], Kay-Tee Khaw [15], Janet L. Stanford [16, 17], William J. Blot [18], Stephen Thibodeau [19], Christiane Maier [20, 21], Adam S. Kibel [22, 23], Cezary Cybulski [24], Lisa Cannon-Albright [25], Hermann Brenner [26], Jong Park [27], Radka Kaneva [28], Jyotsna Batra [29], Manuel R. Teixeira [30], Hardev Pandha[31]

[1] The Institute of Cancer Research, 15 Cotswold Road, Sutton, Surrey, SM2 5NG, UK, [2] Royal Marsden NHS Foundation Trust, Fulham and Sutton, London and Surrey, UK, [3] Centre for Cancer Genetic Epidemiology, Department of Public Health and Primary Care, University of Cambridge, Strangeways Laboratory, Worts Causeway, Cambridge, UK, [4] University of Warwick, Coventry, UK, [5] Cancer Epidemiology Centre, The Cancer Council Victoria, 1 Rathdowne street, Carlton Victoria, Australia, [6] Centre for Molecular, Environmental, Genetic and Analytic Epidemiology, The University of Melbourne, Victoria, Australia, [7] Department of Medical Epidemiology and Biostatistics, Karolinska Institute, Stockholm, Sweden, [8] Department of Preventive Medicine, Keck School of Medicine, University of Southern California/Norris Comprehensive Cancer Center, Los Angeles, California, USA, [9] Department of Medical Biochemistry and Genetics, University of Turku, Turku, Finland, [10] Institute of Biomedical Technology/BioMediTech, University of Tampere and FimLab Laboratories, Tampere, Finland, [11] Department of Clinical Biochemistry, Herlev Hospital, Copenhagen University Hospital, Herlev Ringvej 75, DK-2730 Herlev, Denmark, [12] Cancer Epidemiology Unit, Nuffield Department of Clinical Medicine, University of Oxford, Oxford, UK, [13] Surgical Oncology (Uro-Oncology: S4), University of Cambridge, Box 279, Addenbrooke's Hospital, Hills Road, Cambridge, UK and Cancer Research UK Cambridge Research Institute, Li Ka Shing Centre, Cambridge, UK, [14] Centre for Cancer Genetic Epidemiology, Department of Oncology, University of Cambridge, Strangeways Laboratory, Worts Causeway, Cambridge, UK, [15] Cambridge Institute of Public Health, University of Cambridge, Forvie Site, Robinson Way, Cambridge CB2 0SR, [16] Division of Public Health Sciences, Fred Hutchinson Cancer Research Center, Seattle, Washington, USA, [17] Department of Epidemiology, School of Public Health, University of Washington, Seattle, Washington, USA, [18] International Epidemiology Institute, 1455 Research Blvd., Suite 550, Rockville, MD 20850, [19] Mayo Clinic, Rochester, Minnesota, USA, [20] Department of Urology, University Hospital Ulm, Germany, [21] Institute of Human Genetics University Hospital Ulm, Germany, [22] Brigham and Women's Hospital/Dana-Farber Cancer Institute, 45 Francis Street-ASB II-3, Boston, MA 02115, [23] Washington University, St Louis, Missouri, [24] International Hereditary Cancer Center, Department of Genetics and Pathology, Pomeranian Medical University, Szczecin, Poland, [25] Division of Genetic Epidemiology, Department of Medicine, University of Utah School of Medicine, [26] Division of Clinical Epidemiology and Aging Research, German Cancer Research Center, Heidelberg Germany, [27] Division of Cancer Prevention and Control, H. Lee Moffitt Cancer Center, 12902 Magnolia Dr., Tampa, Florida, USA, [28] Molecular Medicine Center and Department of Medical Chemistry and Biochemistry, Medical University - Sofia, 2 Zdrave St, 1431, Sofia, Bulgaria, [29] Australian Prostate Cancer Research Centre-Qld, Institute of Health and Biomedical Innovation and Schools of Life Science and Public Health, Queensland University of Technology, Brisbane, Australia, [30] Department of Genetics, Portuguese Oncology Institute, Porto, Portugal and Biomedical Sciences Institute (ICBAS), Porto University, Porto, Portugal, [31]The University of Surrey, Guildford, Surrey, GU2 7XH.

Funding for the CRUK study and PRACTICAL consortium:

This work was supported by the Canadian Institutes of Health Research, European Commission's Seventh Framework Programme grant agreement n° 223175 (HEALTH-F2-2009-223175), Cancer Research UK Grants C5047/A7357, C1287/A10118, C5047/A3354, C5047/A10692, C16913/A6135, and The National Institute of Health (NIH) Cancer Post-Cancer GWAS initiative grant: No. 1 U19 CA 148537-01 (the GAME-ON initiative).

COGS acknowledgement:

This study would not have been possible without the contributions of the following: Per Hall (COGS); Douglas F. Easton, Paul Pharoah, Kyriaki Michailidou, Manjeet K. Bolla, Qin Wang (BCAC), Andrew Berchuck (OCAC), Rosalind A. Eeles, Douglas F. Easton, Ali Amin Al Olama, Zsofia Kote-Jarai, Sara Benlloch (PRACTICAL), Georgia Chenevix-Trench, Antonis Antoniou, Lesley McGuffog, Fergus Couch and Ken Offit (CIMBA), Joe Dennis, Alison M. Dunning, Andrew Lee, and Ed Dicks, Craig Luccarini and the staff of the Centre for Genetic Epidemiology Laboratory, Javier Benitez, Anna Gonzalez-Neira and the staff of the CNIO genotyping unit, Jacques Simard and Daniel C. Tessier, Francois Bacot, Daniel Vincent, Sylvie LaBoissière and Frederic Robidoux and the staff of the McGill University and Génome Québec Innovation Centre, Stig E. Bojesen, Sune F. Nielsen, Borge G. Nordestgaard, and the staff of the Copenhagen DNA laboratory, and Julie M. Cunningham, Sharon A. Windebank, Christopher A. Hilker, Jeffrey Meyer and the staff of Mayo Clinic Genotyping Core Facility

Funding for the iCOGS infrastructure came from: the European Community's Seventh Framework Programme under grant agreement n° 223175 (HEALTH-F2-2009-223175) (COGS), Cancer Research UK (C1287/A10118, C1287/A 10710, C12292/A11174, C1281/A12014, C5047/A8384, C5047/A15007, C5047/A10692), the National Institutes of Health (CA128978) and Post-Cancer GWAS initiative (1U19 CA148537, 1U19 CA148065 and 1U19 CA148112 - the GAME-ON initiative), the Department of Defence (W81XWH-10-1-0341), the Canadian Institutes of Health Research (CIHR) for the CIHR Team in Familial Risks of Breast Cancer, Komen Foundation for the Cure, the Breast Cancer Research Foundation, and the Ovarian Cancer Research Fund.

Supplementary table 1. Association of age and PSA at diagnosis, Gleason score and stage of prostate cancer with quartile of polygenic risk score

**Quartile of polygenic risk**	**Late onset (>55 years, n=2632 (%))**	**Early onset (≤55 years, n=106 (%))**	**$\chi 2$ (P value)**
Q1	349 (13)	9 (9)	3.15 (0.369)
Q2	492 (19)	20 (19)	
Q3	754 (29)	28 (26)	
Q4	1037 (39)	49 (46)	
	**Low PSA at diagnosis (≤20 ng/mL, n=2099 (%))**	**High PSA at diagnosis (>20 ng/mL, n=484 (%))**	3.58 (0.311)
Q1	284 (13)	52 (11)	
Q2	396 (19)	93 (19)	
Q3	604 (29)	135 (28)	
Q4	815 (39)	204 (42)	
	**Non-aggressive (Gleason score ≤6, n=1320 (%))**	**Aggressive (Gleason score ≥8, n=368 (%))**	5.37 (0.147)
Q1	168 (13)	64 (17)	
Q2	255 (19)	69 (19)	
Q3	377 (29)	97 (26)	
Q4	520 (39)	138 (38)	
	**Localized stage[1] (n=1879 (%))**	**Advanced stage[2] (n=602 (%))**	1.41 (0.703)
Q1	251 (13)	78 (13)	
Q2	356 (19)	106 (18)	
Q3	544 (29)	169 (28)	
Q4	728 (39)	249 (41)	

p value based on $\chi 2$ test

[1]localized disease is defined as T1-T2, not N1, not M1

[2]advanced disease stage is defined as T3-4, N1, M1

Supplementary table 2. Prostate cancer susceptibility loci (n=55) included in deriving polygenic risk score

Marker	Locus	Alleles[a]	EAF[b]	OR[c] (95% CI)	P_{adj}value[d]
rs16902147	8q24.21	GA	0.07	1.86 (1.56-2.23)	3.525E-8
rs79012498	8q24.21	GA	0.08	1.81 (1.53-2.15)	4.26E-8
rs7832031	8q24.21	AG	0.22	1.49 (1.35-1.65)	3.003E-11
rs2121630	8q24.21	AC	0.16	1.49 (1.33-1.67)	5.691E-8
rs10808558	8q24.21	AG	0.23	1.48 (1.34-1.63)	3.003E-11
rs4314621	8q24.21	GA	0.23	1.48 (1.34-1.63)	3.98E-11
rs7812894	8q24.21	TA	0.23	1.48 (1.34-1.63)	3.694E-11
rs13255059	8q24.21	AG	0.22	1.47 (1.34-1.63)	1.237E-10
rs9297759	8q24.21	AC	0.22	1.46 (1.32-1.61)	4.787E-10
rs11995378	8q24.21	AG	0.22	1.45 (1.32-1.60)	5.568E-10
rs7824868	8q24.21	AG	0.22	1.45 (1.32-1.60)	5.568E-10
rs4242382	8q24.21	AG	0.22	1.45 (1.31-1.60)	1.081E-9
rs4515512	8q24.21	AG	0.22	1.45 (1.31-1.60)	1.081E-9
rs7812429	8q24.21	AG	0.22	1.45 (1.31-1.60)	8.909E-10
rs7814837	8q24.21	AC	0.22	1.45 (1.31-1.60)	9.198E-10
rs10109700	8q24.21	AG	0.23	1.42 (1.29-1.56)	9.646E-9
rs4871801	8q24.21	AG	0.23	1.42 (1.29-1.56)	9.646E-9
rs4871802	8q24.21	CA	0.23	1.42 (1.29-1.56)	9.646E-9
rs6470519	8q24.21	AC	0.23	1.42 (1.29-1.56)	9.646E-9
rs7818556	8q24.21	GA	0.23	1.42 (1.29-1.56)	9.646E-9
rs1447295	8q24.21	AC	0.23	1.41 (1.28-1.55)	2.015E-8
rs4871813	8q24.21	AC	0.22	1.41 (1.28-1.55)	2.332E-8
rs6470529	8q24.21	CG	0.22	1.41 (1.28-1.55)	2.332E-8
rs9643226	8q24.21	GC	0.23	1.41 (1.28-1.55)	1.891E-8
rs1447296	8q24.21	AG	0.23	1.40 (1.27-1.55)	2.603E-8
rs1160267	8p21.2	GA	0.53	1.38 (1.28-1.50)	3.003E-11
rs1512268	8p21.2	AG	0.53	1.38 (1.28-1.50)	3.003E-11
rs995432	8p21.2	GA	0.53	1.38 (1.28-1.50)	3.003E-11
rs13256300	8p21.2	AG	0.51	1.37 (1.27-1.48)	3.003E-11
rs13256366	8p21.2	AG	0.51	1.37 (1.27-1.48)	3.003E-11
rs1398238	8p21.2	CG	0.51	1.37 (1.27-1.48)	3.003E-11
rs1398239	8p21.2	CA	0.51	1.37 (1.27-1.48)	3.003E-11
rs1398240	8p21.2	CA	0.51	1.37 (1.27-1.48)	3.003E-11
rs1512271	8p21.2	AT	0.51	1.37 (1.27-1.48)	3.003E-11
rs2315144	8p21.2	CG	0.51	1.37 (1.27-1.48)	3.003E-11
rs4872171	8p21.2	AG	0.51	1.37 (1.27-1.48)	3.003E-11
rs4872172	8p21.2	AC	0.51	1.37 (1.27-1.48)	3.003E-11
rs4872175	8p21.2	AG	0.53	1.37 (1.27-1.48)	3.003E-11
rs7830220	8p21.2	GA	0.51	1.37 (1.27-1.48)	3.172E-11
rs13265330	8p21.2	GA	0.53	1.37 (1.26-1.48)	4.39E-11
rs7013278	8q24.21	AG	0.38	1.32 (1.21-1.43)	9.387E-8
rs2005705	17q12	AG	0.31	0.76 (0.70-0.82)	8.503E-8
rs10505477	8q24.21	GA	0.46	0.74 (0.69-0.80)	4.785E-10
rs12682374	8q24.21	CG	0.45	0.74 (0.69-0.80)	5.568E-10
rs10486567	7p15.2	AG	0.23	0.74 (0.68-0.81)	6.43E-8
rs67152137	7p15.2	GC	0.23	0.74 (0.68-0.81)	6.43E-8
rs7808935	7p15.2	GA	0.23	0.74 (0.68- 0.81)	6.43E-8
rs6983267	8q24.21	AC	0.43	0.73 (0.68-0.79)	3.172E-11
rs1058205	19p13.33	GA	0.12	0.67 (0.60-0.75)	3.691E-9
rs2569735	19p13.33	AG	0.11	0.67 (0.60-0.75)	2.668E-8
rs2735839	19p13.33	AG	0.11	0.67 (0.60-0.75)	2.668E-8
rs174776	19p13.33	AG	0.07	0.63 (0.55-0.71)	2.307E-8
rs266878	19p13.33	CG	0.07	0.62 (0.54-0.71)	2.332E-8
rs17632542	19p13.33	GA	0.06	0.59 (0.51-0.68)	3.015E-9
rs62113212	19p13.33	AG	0.06	0.59 (0.51-0.68)	2.846E-9

[a]Effect allele/Other allele; [b]Effect allele frequency in cases; [c]Per-allele odds ratio for the effect allele
[d]Adjusted for false discovery rate (FDR) using Benjamini-Hochberg method;
